# Polymers and Chemical Composition of Hardwood and Softwood (Bark, Sapwood, and Heartwood) for Biofuel Production: A Comprehensive Review

**DOI:** 10.3390/polym18111340

**Published:** 2026-05-28

**Authors:** Ria Aniza, Anelie Petrissans, Mathieu Petrissans

**Affiliations:** 1 Laboratoire d’Etudes et de Recherche sur le Matériau Bois, Institut National de Recherche pour l’Agriculture, l’Alimentation et l’Environnement (INRAE), Université de Lorraine, F88000 Epinal, France; mathieu.petrissans@univ-lorraine.fr; 2 International Doctoral Degree Program in Energy Engineering, National Cheng Kung University, Tainan 701, Taiwan

**Keywords:** lignocellulosic polymers, hemicellulose–cellulose–lignin, biomass fractionation, resource valorization, bio-based production

## Abstract

Lignocellulosic biomass from hardwood and softwood species represents a highly abundant and renewable resource for biofuel and bio-based material production. This review provides a comprehensive analysis of the chemical composition and structural organization of the three major polymers—hemicellulose, cellulose, and lignin—across different wood fractions, including bark, sapwood, and heartwood. Typically, wood consists of a significant number of these components, approximately 20–35% hemicellulose, 40–50% cellulose, and 20–30% lignin. Significant variations exist between hardwood and softwood species, particularly in lignin composition and hemicellulose structure, which strongly influence biomass recalcitrance and conversion efficiency. Bark is rich in lignin (often 20–40%) and extractives, making it suitable for thermochemical processes, while sapwood exhibits higher carbohydrate accessibility, favoring biochemical conversion. Heartwood, enriched with extractives and condensed lignin, shows reduced reactivity but high potential for value-added chemicals. The review also evaluates extraction techniques and conversion pathways, highlighting the importance of fraction-specific processing strategies. Understanding these variations is essential for optimizing biorefinery performance and advancing sustainable biomass utilization.

## 1. Introduction

**Figure 1:**
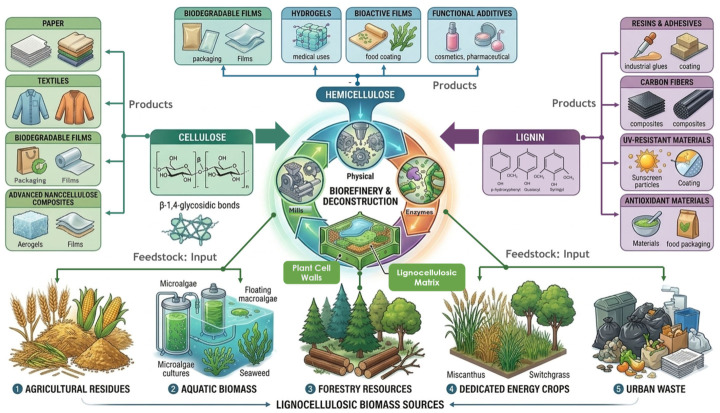
The lignocellulosic biorefinery ecosystem: A schematic overview of biomass sources and the value-added products derived from cellulose, hemicellulose, and lignin.

Lignocellulosic biomass is one of the most abundant and renewable feedstocks on Earth, offering considerable potential for the production of high-value polymers. It is primarily composed of three major polymers (Figure 1)—hemicellulose, cellulose, and lignin—which form a complex, hierarchical, and recalcitrant matrix that provides mechanical strength and biological resistance to plant tissues [1,2]. These components are increasingly exploited in diverse industrial applications: hemicelluloses are utilized in hydrogels, bioactive films, biodegradable films, and functional additives; cellulose is widely used in paper, textiles, biodegradable films, and advanced nanocellulose composites; and lignin is gaining attention as a renewable precursor for resins and adhesives, carbon fibers, UV-resistant materials, and antioxidant materials [3,4]. Lignocellulosic biopolymers can be derived from various biomass sources, including agricultural residues [5,6], aquatic biomass such as micro- and macroalgae [7], forestry resources [8,9], dedicated energy crops [10,11], and urban waste [11,12].

The continued reliance on fossil fuels for energy production remains a major environmental concern, as their non-renewable nature and combustion contribute significantly to air pollution, including the release of hazardous compounds such as dioxins and furans [13,14]. Although lignocellulosic biomass is considered a renewable and sustainable alternative to fossil resources, biomass pretreatment and thermochemical processing may also generate inhibitory and potentially toxic compounds such as furfural and 5-hydroxymethylfurfural (HMF) through carbohydrate dehydration reactions. These furanic aldehydes can negatively affect downstream fermentation and biofuel production efficiency [15,16]. Furfural (typically 0.1–5 g/L) and HMF (0.1–3 g/L), generated during lignocellulosic pretreatment, can inhibit microbial fermentation at concentrations as low as ~0.5–1 g/L, leading to ethanol yield reductions of up to 10–70% depending on process conditions [16,17]. These limitations do not outweigh the overall advantages of lignocellulosic biomass as a sustainable and widely available resource. Continued improvements in pretreatment and bioprocess engineering are helping to reduce the impact of these compounds. Moreover, the chemical composition and structural characteristics of woody lignocellulosic polymers vary considerably between hardwood and softwood species [1,18], as well as among different anatomical regions such as sapwood, heartwood, and bark. These variations strongly influence their extractability and suitability for specific applications. A thorough understanding of such differences is therefore essential for optimizing extraction processes, improving polymer performance, and advancing the development of sustainable, bio-based materials for the polymer industry [19,20].

Globally, softwood accounts for approximately 70–75% of industrial lumber production, while hardwood represents about 25–30%, although total harvesting of hardwood and softwood biomass is more balanced due to significant hardwood use in fuel and non-industrial applications [21]. Based on their fiber composition, hardwoods (angiosperms) and softwoods (gymnosperms) exhibit notable differences in their lignocellulosic composition and polymer structures. In contrast to cellulose and hemicellulose—which are complex polysaccharides—lignin is a structurally distinct polymer that is a highly complex, three-dimensional aromatic network formed from phenolic monomers. Hardwood lignin (Figure 1) typically contains both guaiacyl (G) and syringyl (S) units, whereas softwood lignin is predominantly composed of guaiacyl units [22,23]. Additionally, the main anatomical fractions of woody biomass—sapwood, heartwood, and bark—differ significantly in terms of polymer composition, extractives content, and microstructure. These variations strongly influence extraction efficiency and determine the suitability of each fraction for specific applications. To recover lignocellulosic polymers or convert biomass into energy products such as heat, biochar, bio-oil, and syngas, numerous studies have explored integrated thermochemical conversion pathways. These include processes such as torrefaction, pyrolysis, combustion, hydrothermal liquefaction (HTL), and gasification [12,24,25].

In woody biomass, typically, the ease of extraction of the three principal lignocellulosic polymers generally follows the order: hemicellulose < lignin < cellulose, corresponding to increasing structural resistance. Typically, woody biomass consists of approximately 40–50% cellulose, 20–35% hemicellulose, and 20–30% lignin, depending on species, growth conditions, and processing methods [26,27]. Hemicellulose is the most readily extractable fraction due to its amorphous, branched structure and relatively low molecular weight. It is only weakly associated with cellulose and lignin within the cell wall matrix and can be efficiently hydrolyzed using dilute acids, alkaline solutions, or hot water [10]. Lignin exhibits intermediate resistance, as it is a complex, three-dimensional aromatic polymer with extensive crosslinking. Although more recalcitrant than hemicellulose, lignin can be partially removed through chemical treatments such as alkaline pulping or organic solvent (organosolv) processes. In contrast, cellulose is the most resistant component, owing to its highly ordered, crystalline structure and strong intermolecular hydrogen-bonding network, which necessitate harsh chemical or enzymatic pretreatment for effective extraction.

In contrast, the thermal resistance of wood polymers follows the order hemicellulose < cellulose < lignin due to differences in their molecular structures. Hemicellulose exhibits both the lowest extractability resistance and the lowest thermal stability, decomposing first at approximately 200–300 °C due to its amorphous, branched structure and labile functional groups [28]. Cellulose shows intermediate thermal resistance, with major decomposition occurring around 300–400 °C, attributed to its linear polymer chains and strong intermolecular hydrogen bonding that promote high crystallinity and stability [29]. Lignin displays the highest thermal resistance, degrading slowly over a broad range of about 250–500 °C and even higher depending on conditions due to its complex, highly crosslinked aromatic structure [8]. As a result, lignin contributes most significantly to char formation during thermal conversion processes of lignocellulosic biomass.

Most existing reviews on lignocellulosic biomass focus broadly on whole-wood composition or general extraction technologies, with limited attention to a systematic comparison of anatomical fractions such as sapwood, heartwood, and bark across both hardwood and softwood species. In particular, there remains a lack of integration between fraction-specific polymer characteristics and their implications for downstream applications, especially in biofuel and bio-based material production. This gap is increasingly critical as the demand for efficient biomass utilization intensifies under the global transition toward renewable energy and carbon-neutral systems. This review addresses this limitation by critically synthesizing current knowledge on the chemical and structural features of lignocellulosic polymers in different wood fractions, alongside their extraction strategies, conversion pathways, and application potential. The novelty of this work lies in its fraction-resolved perspective, linking anatomical variability directly to process performance and end-use functionality. The urgency of this topic is underscored by the need to improve biomass valorization efficiency and reduce reliance on fossil resources. This review will benefit researchers in biomass conversion, chemical engineering, materials science, and bioenergy sectors, as well as industrial stakeholders developing sustainable biorefinery technologies.

## 2. Woody Biomass and Lignocellulosic Polymer Composition

**Figure 2:**
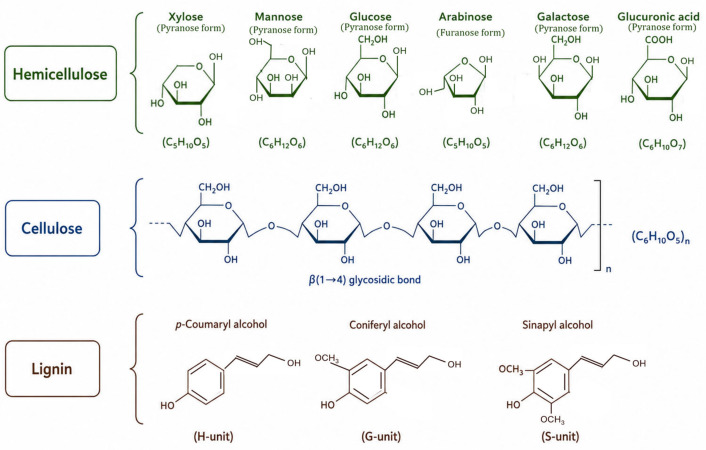
Schematic representation of the structural and chemical complexity of lignocellulosic biomass components—Hemicellulose, Cellulose, and Lignin.

Woody biomass is primarily composed of three lignocellulosic polymers (Figure 2)—hemicellulose, cellulose, and lignin—along with smaller amounts of extractives and inorganic minerals [1,9]. The relative proportions and structural organization of these components vary with species, growth conditions, and anatomical regions such as bark, sapwood, and heartwood. These variations strongly influence biomass reactivity, processing behavior, pulping efficiency, and biofuel conversion performance.

Hemicellulose (Figure 2) is a heterogeneous group of amorphous, branched polysaccharides composed of various sugar monomers, including xylose (C_5_H_10_O_5_), mannose (C_6_H_12_O_6_), glucose (C_6_H_12_O_6_), arabinose (C_5_H_10_O_5_), galactose (C_6_H_12_O_6_), and uronic acids (typically C_6_H_10_O_7_) such as glucuronic acid [27,30]. Although some monosaccharides, such as xylose and arabinose, share the same molecular formula (C_5_H_10_O_5_), they are structural isomers, specifically stereoisomers (epimers), that differ in the spatial configuration of hydroxyl (–OH) groups around their chiral carbon atoms. Unlike cellulose, it lacks a uniform repeating structure and is highly species-dependent. Major structural types include glucuronoxylans (hardwoods), galactoglucomannans (softwoods), and arabinoxylans (grasses and some woody tissues). Functionally, hemicellulose acts as a matrix polymer that binds cellulose microfibrils and interacts closely with lignin, contributing to structural flexibility and moisture regulation. Cellulose (Table 1) is a linear homopolysaccharide consisting of repeating β-D-glucopyranose units linked by β(1→4) glycosidic bonds, with the general empirical formula of monomer glucose (C_6_H_10_O_5_)ₙ [31,32]. Its fundamental repeating unit is cellobiose (C_12_H_22_O_11_), a glucose dimer linked by a β(1→4) glycosidic bonds—is commonly produced during the hydrolysis of cellulose. Cellulose forms the primary structural framework of lignocellulosic biomass, assembling into highly ordered microfibrils stabilized by extensive hydrogen bonding, which provides mechanical strength and rigidity. Moreover, lignin is a complex, three-dimensional phenylpropanoid polymer derived from three monolignols: p-coumaryl alcohol (H units), coniferyl alcohol (G units), and sinapyl alcohol (S units) [22,27]. These units are interconnected through ether and carbon–carbon linkages, including β–O–4 (dominant), β–β, β–5, and 5–5 bonds. Lignin composition varies among biomass types, including softwoods that are predominantly composed of guaiacyl (G) units, hardwoods that contain both syringyl (S) and guaiacyl (G) units, and grasses that incorporate all three units, namely p-hydroxyphenyl (H), guaiacyl (G), and syringyl (S).

Together, hemicellulose, cellulose, and lignin (Table 1) form an integrated and hierarchically organized network whose composition and interactions determine the physicochemical and conversion properties of woody biomass. Importantly, these polymers are not uniformly distributed across the tree structure; instead, their abundance and accessibility differ significantly between bark, sapwood, and heartwood, as well as between hardwood and softwood species. Therefore, a detailed understanding of anatomical variation is essential for accurate assessment of biomass fractionation, pretreatment behavior, and biofuel conversion efficiency. In the following sections, the compositional and structural characteristics of hardwood bark, sapwood, and heartwood are examined with respect to their implications for biomass processing and biofuel production, followed by a comparative discussion of the corresponding softwood fractions to highlight differences in lignocellulosic organization and utilization potential.

**Figure 3:**
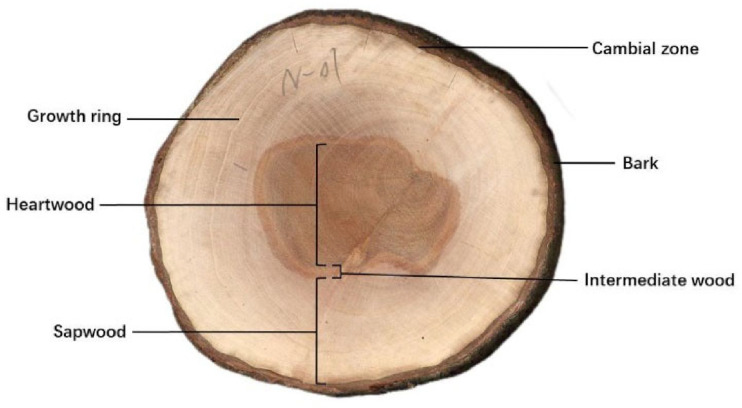
Illustration of the spatial distribution of bark, sapwood, and heartwood in a cross-section of sandalwood (a hardwood species) [35]. Adapted with permission from Ref. [35]. Life, 2025, License under CC BY 4.0.

Bark, sapwood, and heartwood are typically separated during wood processing based on their anatomical and physical differences [33,34]. In both hardwood and softwood species, bark is first removed mechanically through a debarking process using drum, ring, or hydraulic debarkers. After debarking, the logs are cut to expose the internal wood regions. Sapwood and heartwood are then distinguished based on differences in color, moisture content, density, and chemical composition. Heartwood is generally darker, denser, and richer in extractives, whereas sapwood is lighter in color and characterized by higher moisture content. In hardwood species (e.g., sandalwood, as shown in Figure 3), the boundary between sapwood and heartwood is often visually distinct due to pronounced color differences, allowing for manual or mechanical separation during sawing [35]. In softwoods, however, this boundary is frequently less defined, and separation may require additional approaches such as moisture content analysis, staining techniques, or image-based detection systems. At the industrial scale, logs are commonly sawn into sections in which heartwood and sapwood can be selectively processed. In laboratory or research settings, these fractions are typically isolated manually using knives, chisels, or precision cutting tools to ensure accurate and reproducible sampling. 

### 2.1. Hardwood: Bark, Sapwood, and Heartwood

**Figure 4:**
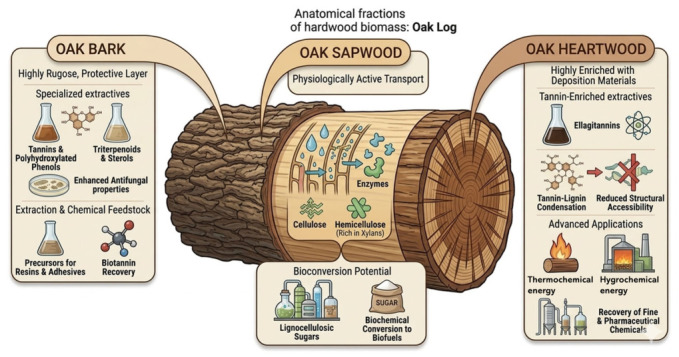
Compositional Mapping of Oak Log Sections: A comparative overview of bark, sapwood, and heartwood.

Hardwood species such as oak, beech, poplar, birch, eucalyptus, maple, acacia, aspen, and willow are widely distributed across temperate, subtropical, and tropical regions, depending on the species and climate adaptability. These species exhibit distinct anatomical and chemical variations among bark, sapwood, and heartwood. Each wood fraction differs in biological function, developmental stage, extractive content, and lignocellulosic polymer composition, which collectively govern its physicochemical properties and suitability for industrial applications. Using hardwood species such as oak wood as an example (Figure 4), bark, sapwood, and heartwood exhibit distinct structural and chemical characteristics that reflect their specific physiological roles and maturation processes.

Understanding these fraction-dependent variations is therefore essential for optimizing biomass utilization in material development, bioenergy production, and integrated biorefinery systems (Table 2).

#### 2.1.1. Hardwood Bark

Bark is the outer protective tissue of hardwood stems and branches, comprising inner bark (phloem) and outer bark (rhytidome). It functions as a defensive barrier against mechanical damage, pathogens, moisture loss, and environmental stress. Compared with internal wood tissues, hardwood bark exhibits a highly heterogeneous structure and chemical composition characterized by lower structural carbohydrates and higher levels of lignin, extractives, and inorganic minerals [41]. From a lignocellulosic perspective, cellulose content in hardwood bark is relatively low and exists in a less ordered microfibrillar form embedded within a dense matrix of lignin and extractives, limiting its accessibility [37]. Hemicellulose is highly variable and more branched than in wood tissues, commonly including glucuronoxylans along with arabinose-, galactose-, and uronic acid-rich polysaccharides, which contribute to water-binding capacity and structural flexibility [27,47]. Lignin in bark is generally more condensed and chemically heterogeneous, often associated with phenolic compounds and suberin-like aliphatic structures, resulting in increased resistance to chemical and biological degradation [41,57].

Hardwood bark (Table 3) is characterized by a high concentration of extractives, typically accounting for approximately 5–30% of dry mass depending on species, age, and growth conditions [41,57]. These extractives include tannins, flavonoids, phenolic acids, waxes, resins, and suberin-derived compounds, which contribute to properties such as hydrophobicity, antimicrobial resistance, pigmentation, and natural durability. In some species, tannins alone may constitute up to 10–20% of bark dry weight [58]. However, these compounds can inhibit microbial fermentation and enzymatic conversion, although they also provide opportunities for high-value chemical recovery. Bark generally contains higher ash content (~2–10%) compared to wood (<1%) bark, which significantly affects combustion and thermochemical behavior. Marked interspecific variability is observed in bark composition. For example, *Quercus* spp. (oak) bark contains high lignin levels (~25–40%) and significant tannin content; *Betula* spp. (birch) bark is enriched with triterpenoids such as botulin, reaching 10–15% of dry mass [59]; *Eucalyptus* spp. exhibits variable lignin (~20–35%) depending on species and site conditions; *Acer* spp. (maple) shows a more balanced carbohydrate profile with moderate lignification [60], whereas *Populus* spp. (poplar) typically contains lower lignin (~18–25%) and higher polysaccharide accessibility, making it more suitable for biochemical conversion. 

In biofuel applications, hardwood bark is generally more suitable for thermochemical and integrated biorefinery or soil nourishment pathways than for conventional pulping processes. Its relatively high lignin content (often ~20–40%) enhances its suitability for energy-dense product formation via pyrolysis, gasification, and combustion, leading to the production of bio-oil, syngas, and biochar. In parallel, the hemicellulose and cellulose fractions can be depolymerized into fermentable sugars through appropriate pretreatment strategies, such as dilute acid, alkaline treatment, or steam explosion, enabling subsequent fermentation to bioethanol and other advanced biofuels. However, the presence of abundant extractives may generate inhibitory compounds, often requiring additional detoxification or conditioning steps prior to biochemical conversion. Hardwood bark represents a structurally complex but chemically valuable biomass fraction. While its high lignin and extractive contents limit its suitability for fiber-based applications, these same characteristics make it a promising feedstock for bioenergy production, biofuel synthesis, and high-value biochemical recovery within integrated biorefinery systems.

#### 2.1.2. Hardwood Sapwood

Hardwood sapwood is the physiologically active outer region of the stem responsible for water transport and nutrient storage. Compared with heartwood and bark, it is more chemically uniform, contains lower levels of extractives, and is more accessible to chemical and enzymatic treatments, making it a highly suitable fraction of lignocellulosic biomass for biofuel production. Sapwood is primarily composed of hemicellulose, cellulose, and lignin, with minor amounts of extractives and inorganic constituents. Cellulose is the dominant structural polymer, forming crystalline microfibrils that provide mechanical strength, and its accessibility makes it a crucial source of fermentable glucose after enzymatic hydrolysis [61,62]. Hemicellulose in hardwood sapwood is mainly O-acetyl-4-O-methylglucuronoxylan, which is more readily hydrolyzed than cellulose to yield xylose and other fermentable sugars. Lignin, present at moderate levels (~20–25%), consists mainly of syringyl (S) and guaiacyl (G) units and is generally less condensed in sapwood, improving delignification efficiency and enzymatic digestibility [26,63]. Extractives are relatively low and include minor fatty acids, resins, and phenolic compounds, which further reduce fermentation inhibition and enhance microbial conversion efficiency. 

The composition of sapwood makes it suitable for both biochemical and thermochemical conversion pathways. In biochemical processes, pretreatments such as steam explosion, dilute acid, alkaline, and organosolv methods disrupt the lignin–carbohydrate matrix, enabling enzymatic hydrolysis of cellulose and hemicellulose into fermentable sugars for bioethanol, biobutanol, and other bio-based chemicals [45,47]. In thermochemical conversion, sapwood can be processed via pyrolysis, gasification, or hydrothermal liquefaction to produce bio-oil, syngas, and biochar. Its relatively low extractive and ash content contributes to more stable conversion behavior compared with bark-rich feedstocks.

#### 2.1.3. Hardwood Heartwood

Heartwood is formed through the progressive transformation of sapwood, during which living parenchyma cells lose metabolic activity, and the tissue becomes enriched with deposited extractives and chemically modified polymers. In hardwood species, heartwood typically accounts for a substantial portion of stem volume and is characterized by higher density, darker coloration, and biological inactivity, functioning mainly as structural support rather than a conductive tissue. From a compositional perspective, hardwood heartwood still contains the main lignocellulosic polymers—cellulose (~40–45%), hemicellulose (~15–25%), and lignin (~25–35%)—but their accessibility is significantly reduced due to pore blockage and chemical modification by extractives and lignin condensation [45,47,63]. Cellulose content remains relatively unchanged compared with sapwood; however, its accessibility decreases due to impregnation by hydrophobic compounds and reduced porosity. Hemicellulose (mainly O-acetyl-4-O-methylglucuronoxylan in hardwoods) is more strongly associated with lignin and extractives, resulting in lower enzymatic hydrolysis efficiency and reduced fermentable sugar yields. Lignin content is often slightly higher or more condensed (~25–35%) than in sapwood, with increased cross-linking and oxidative aging, which reduces reactivity and increases hydrophobicity.

A defining feature of hardwood heartwood is its high extractive content, typically ~5–30% of dry mass, including phenolics, tannins, quinones, flavonoids, resins, fatty acids, and terpenoids [35,64]. These compounds fill cell lumens and restrict diffusion pathways, enhancing natural durability but limiting chemical penetration and enzymatic accessibility. Consequently, hardwood heartwood is less suitable for biochemical conversion, often requiring more severe pretreatment conditions to achieve effective delignification and sugar release. In comparison, softwood heartwood differs significantly: it is generally richer in condensed guaiacyl (G-type) lignin (~27–35%), contains higher resin and resin acid content (up to 10–15% in some species), and lacks syringyl (S) units, resulting in a more condensed and less reactive lignin network [63,64]. Softwood heartwood is therefore typically more recalcitrant than hardwood heartwood, showing lower delignification efficiency and higher resistance to enzymatic hydrolysis due to its highly crosslinked G-lignin structure.

Despite these limitations, heartwood remains a valuable source of bioactive compounds, as its extractives exhibit antioxidant, antimicrobial, and preservative properties suitable for pharmaceuticals and specialty chemicals. In thermochemical conversion processes such as pyrolysis and gasification, heartwood generally yields higher proportions of aromatic compounds and biochar due to its elevated lignin and extractive content. Heartwood represents a chemically transformed, extractive-rich biomass fraction with reduced biochemical reactivity but enhanced potential for high-value chemical recovery and carbon-rich material production.

### 2.2. Softwood: Bark, Sapwood, and Heartwood

**Figure 5:**
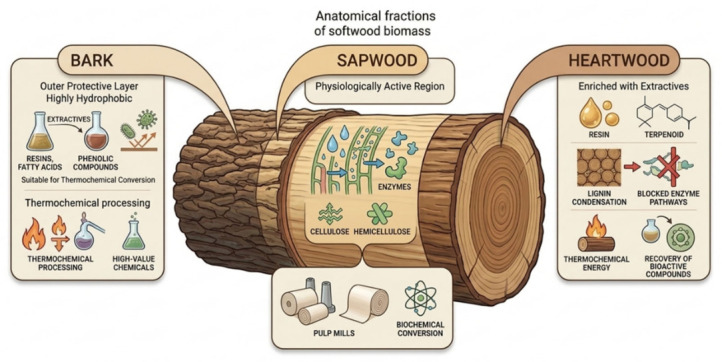
Compositional Mapping of Pine Log Sections: A comparative overview of bark, sapwood, and heartwood.

Softwoods differ from hardwoods in both anatomical structure and lignocellulosic polymer composition (Figure 5). Unlike hardwoods, which contain vessels and fibers, softwoods are composed almost exclusively of tracheids, resulting in a more uniform and less porous cellular structure that typically constitutes ~90–95% of the wood volume. This anatomical simplicity strongly influences polymer distribution and accessibility within bark, sapwood, and heartwood. In terms of composition, softwoods generally contain higher lignin content (~25–31%) than hardwoods (~20–25%), while cellulose remains relatively similar at ~40–45% in both groups [1,65]. Hemicellulose composition also differs, with softwoods being rich in galactoglucomannan and arabinoglucuronoxylan, whereas hardwoods are dominated by xylan-based hemicellulose. Extractives in softwoods typically range from ~1–10%, including resins, terpenes, and fatty acids, which can be higher in heartwood regions and significantly influence processing behavior [66].

These structural and chemical differences make softwoods generally more recalcitrant than hardwoods in bioconversion processes. The higher proportion of condensed guaiacyl (G-type) lignin in softwoods contributes to stronger cross-linking and reduced chemical reactivity, thereby increasing resistance to pulping, enzymatic hydrolysis, and thermochemical deconstruction [65,67]. As a result, softwood fractions—particularly heartwood—often require more severe pretreatment conditions compared to hardwoods to achieve comparable levels of delignification and carbohydrate accessibility. Although the genus is included in the Gymnospermae, the polymers in softwood may diverge because of several factors, including the climate zone and region where the woods are habitat (Table 4).

#### 2.2.1. Softwood Bark

Softwood bark forms the outermost protective tissue of gymnosperm trees, functioning as a barrier against mechanical damage, pathogens, desiccation, and environmental stress. It consists of inner bark (secondary phloem) and outer bark (rhytidome), which includes successive layers of dead protective tissues. Compared with the underlying wood, softwood bark is chemically heterogeneous and structurally complex, with composition varying among species and growth conditions. From a lignocellulosic perspective, softwood bark contains lower amounts of structural polysaccharides than sapwood or heartwood, with cellulose content typically ranging from 18% to 30% [68,82]. The existing cellulose is less accessible due to its embedding within a dense matrix of lignin and extractives. Hemicellulose in softwood bark is highly heterogeneous, often enriched with arabinogalactans and pectic substances, making up roughly 15% to 25% of the dry mass [82,83]. These polymers contribute to structural irregularity and variable reactivity during chemical processing.

Lignin content in softwood bark is relatively high, often between 25% and 55% (including “bark phenolic acids”), and is typically more condensed than wood lignin. This lignin-rich structure enhances rigidity and resistance to microbial degradation while reducing susceptibility to enzymatic hydrolysis. In addition to structural polymers, softwood bark contains substantial amounts of extractives (15–30%), including resin acids, terpenoids, and suberin-related aliphatic components. Suberin, in particular, can account for up to 2% to 10% of the dry weight in certain species, imparting hydrophobicity and antimicrobial properties [82,84].

Softwood bark also exhibits higher ash and mineral content (1.5% to 5.0%) than internal wood fractions (<0.5%), including calcium, potassium, and silica [69,80,81]. These inorganic constituents increase slagging or fouling tendencies during thermochemical processing. While generally unsuitable for conventional pulp production, the chemical richness of softwood bark makes it an attractive feedstock for biorefineries. It is commonly utilized for pyrolysis and gasification to produce bio-oil and syngas, while its extractive fraction can be valorized for tannins and antioxidants. Softwood bark is better suited for thermochemical conversion and chemical recovery than for fiber-based or sugar-platform biofuel production.

#### 2.2.2. Softwood Sapwood

Softwood sapwood represents the outer, physiologically active region of the xylem, responsible for water transport and nutrient storage, and is typically characterized by lighter color, higher moisture content, and greater permeability than heartwood [22,72]. This makes it a relatively accessible and favorable feedstock for biorefinery and industrial applications. Chemically, softwood sapwood is dominated by lignocellulosic polymers, with cellulose accounting for approximately 40–45% of dry mass, forming the primary structural framework of the cell wall. Hemicellulose content ranges from 20–30%, with galactoglucomannan (15–20%) as the major component and arabinoglucuronoxylan (5–10%) as a secondary fraction. Lignin typically constitutes 26–34%, composed predominantly of guaiacyl (G) units, resulting in a more condensed and recalcitrant three-dimensional structure compared to hardwood lignin [69,81]. Extractives are relatively low (~2–5%), contributing to high permeability due to the absence of resin accumulation and vessel blockage, which facilitates chemical dispersion.

These characteristics make softwood sapwood suitable for multiple conversion pathways. In biochemical processes, its low extractive content and relatively high carbohydrate availability enhance enzymatic saccharification and fermentation efficiency, although lignin recalcitrance remains a limiting factor. In pulping and fiber applications, the long tracheid fibers and high cellulose content provide superior mechanical strength, making softwood sapwood a preferred raw material for the paper and textile industries. Additionally, its permeability and reduced chemical barriers improve pretreatment efficiency, leading to higher sugar yields during saccharification and subsequent bioethanol production.

#### 2.2.3. Softwood Heartwood

Softwood heartwood forms as sapwood undergoes complex physiological and biochemical transformations during tree maturation, a process characterized by the intensive deposition of secondary metabolites. This transition involves the accumulation of extractives—such as resin acids, stilbenes, lignans, and phenolic compounds—which enhance the wood’s natural durability and resistance to biological decay, often resulting in a darker color [23,68]. Chemically, while cellulose and hemicellulose contents remain relatively stable at approximately 40% to 45% and 20% to 30%, respectively, their accessibility is significantly reduced due to extractive deposition and the physical barrier of pit aspiration. Furthermore, heartwood typically exhibits a slightly higher lignin concentration, ranging from 26% to 34%, with a more condensed chemical structure that further increases resistance to chemical and microbial degradation [76,77]. These characteristics lead to reduced permeability and decreased enzymatic hydrolysis rates, making heartwood less efficient for pulping or biofuel production compared to sapwood, though highly valued for structural and outdoor applications.

Softwood bark, sapwood, and heartwood represent three distinct chemical environments that dictate their industrial utility. Bark is a lignin- and extractive-rich fraction with relatively low carbohydrate content, making it best suited for thermochemical conversion and chemical recovery. Sapwood provides the highest accessibility to structural carbohydrates and the lowest extractive levels (2% to 5%), making it the optimal fraction for biochemical conversion and high-strength fiber applications. Conversely, heartwood is defined by its enrichment of phenolic compounds and reduced permeability, which increases its recalcitrance to processing but enhances its natural durability. Understanding these radial variations is essential for determining the most effective processing strategies and end-use applications for softwood lignocellulosic biomass.

In general, hemicellulose, cellulose, and lignin make up the majority of woody biomass polymers, and their relative abundance and structural arrangement have a significant impact on biomass reactivity and conversion efficiency. Lignin increases rigidity and resistance to degradation, hemicellulose adds flexibility and interpolymer interactions, and cellulose’s crystalline structure gives it mechanical strength. Hardwoods and softwoods, as well as the portions of bark, sapwood, and heartwood, differ significantly in composition. Hardwood lignin is more recalcitrant because it contains both syringyl and guaiacyl units, while softwood lignin is primarily guaiacyl-based. Optimizing pretreatment, extraction, and sustainable biofuel production processes requires a consideration of these lignocellulosic properties.

## 3. Extraction Strategies for Lignocellulosic Polymers

### 3.1. Hardwood Polymers Extraction

Hardwood biomass—derived from deciduous species such as birch (*Betula* spp.), poplar (*Populus* spp.), and eucalyptus (*Eucalyptus* spp.)—presents a structurally complex yet comparatively accessible matrix for polymer recovery. Its cell walls are primarily composed of cellulose (~40–50%), hemicellulose (~20–30%), and lignin (~18–25%), with a lignin structure enriched in both syringyl (S) and guaiacyl (G) units. This higher S/G ratio generally results in a less condensed lignin network than that of softwoods, facilitating chemical delignification and enhancing enzymatic accessibility [27,48]. A significant feature of hardwoods is the abundance of glucuronoxylan, a major hemicellulosic component accounting for approximately 15–30% of dry weight. While this polymer contributes to structural integrity, it also introduces challenges during extraction due to its acetylation and interactions with lignin, which can generate inhibitory compounds during pretreatment.

The recovery of hemicellulose, cellulose, and lignin from hardwood biomass is typically achieved through a combination of physicochemical and biochemical processes. Hemicellulose is the most readily extractable fraction and can be selectively solubilized using hot water, dilute acid hydrolysis, or alkaline extraction. Cellulose, being highly crystalline and structurally robust, requires more intensive pretreatment—such as steam explosion, organosolv, or alkaline delignification—to disrupt the lignin–carbohydrate matrix before enzymatic hydrolysis into fermentable sugars. Lignin can be isolated through processes such as kraft pulping, sulfite treatment, or organosolv fractionation, yielding a reactive aromatic polymer suitable for further valorization. The relatively lower lignin content and more labile lignin structure of hardwoods make them particularly attractive for integrated biorefinery applications.

#### 3.1.1. Hardwood Hemicellulose

Because hardwoods are rich in xylan-type hemicellulose, particularly O-acetyl-4-O-methylglucuronoxylan, the most widely applied industrial strategy for its recovery is hot water extraction (HWE) or autohydrolysis [45,47]. This process exploits the naturally occurring acetyl groups in hardwood hemicellulose. Under elevated temperatures (typically 160–200 °C) and autogenous pressure, these acetyl groups are cleaved, releasing acetic acid, which lowers the pH to around 3–4 and promotes autocatalytic hydrolysis of hemicellulose into soluble oligosaccharides and monomeric sugars. As a result, a significant fraction of hemicellulose (~50–80% removal, depending on conditions) can be selectively solubilized while largely preserving the cellulose-rich fiber matrix [30,47].

This approach is considered environmentally favorable because it avoids the use of strong mineral acids or bases, reduces chemical consumption, and minimizes downstream neutralization requirements. In addition, autohydrolysis enhances fiber porosity and improves the efficiency of subsequent delignification and enzymatic hydrolysis steps, making it particularly attractive for integrated biorefinery systems [27,62]. However, process severity must be carefully controlled, as excessive degradation can lead to the formation of inhibitory byproducts such as furfural and acetic acid, which can negatively impact fermentation performance. Overall, hot water extraction provides a selective, scalable, and sustainable route for hemicellulose recovery from hardwood biomass while maintaining the integrity of cellulose for further valorization.

#### 3.1.2. Hardwood Cellulose

Cellulose extraction from hardwood biomass typically involves a sequence of pretreatment and delignification steps designed to disrupt the tightly bound lignin–carbohydrate matrix and expose the crystalline cellulose microfibrils. After partial removal of hemicellulose (e.g., via autohydrolysis) [48], cellulose is enriched through chemical pulping or fractionation processes. The most established industrial method is kraft pulping, which uses sodium hydroxide and sodium sulfide at temperatures of approximately 150–170 °C to solubilize lignin and part of the hemicellulose, yielding a cellulose-rich pulp that typically represents ~40–55% of the original dry biomass. Alternative approaches such as organosolv pretreatment (e.g., ethanol–water systems at 160–200 °C) or alkaline extraction can produce cellulose with lower residual lignin content and improved purity, making it more suitable for downstream applications such as nanocellulose production or enzymatic conversion [37,47].

Despite its high crystallinity and resistance to chemical attack, cellulose becomes more accessible after pretreatment due to the removal of surrounding lignin and hemicellulose and the increase in pore volume. This enhanced accessibility enables efficient enzymatic hydrolysis, where cellulases convert cellulose into glucose with conversion efficiencies often reaching 80–90% under optimized conditions [85]. The resulting glucose can be fermented into bioethanol or other bio-based chemicals. In addition, extracted cellulose can be further processed into high-value materials such as cellulose nanofibers, films, and biodegradable polymers.

#### 3.1.3. Hardwood Lignin

Lignin extraction from hardwood biomass is typically performed concurrently with or following cellulose isolation, as lignin forms the primary structural barrier limiting biomass deconstruction. In conventional kraft pulping, lignin is cleaved and solubilized into the alkaline liquor (black liquor), from which it can be recovered by acidification and precipitation [40,46]. Although this process is highly efficient for delignification, the recovered lignin is often chemically modified (e.g., sulfur-containing and partially condensed), which can limit its reactivity for certain high-value applications. In contrast, organosolv processes employ organic solvents (such as ethanol or acetone) under elevated temperatures (~160–200 °C) to extract lignin in a more native-like form, yielding sulfur-free lignin with relatively preserved functional groups and a narrower molecular weight distribution [55,62]. Typical lignin recovery yields range from ~50–70% of the original lignin content, depending on process conditions.

The extracted lignin, due to its aromatic and highly crosslinked structure, is increasingly recognized as a valuable renewable resource. It can be upgraded into phenolic resins, adhesives, polyurethane precursors, and carbon fibers, or depolymerized into platform chemicals such as vanillin and phenols. In thermochemical conversion pathways, lignin contributes significantly to biochar formation and aromatic-rich bio-oil, reflecting its high thermal stability and carbon content. However, efficient lignin extraction requires careful control of process severity to avoid excessive condensation reactions, which reduce solubility and downstream reactivity. Consequently, modern biorefinery strategies aim to integrate lignin-first or fractionation approaches that preserve lignin quality while maximizing overall biomass valorization.

### 3.2. Softwood Polymers Extraction

#### 3.2.1. Softwood Hemicellulose

Hemicellulose extraction from softwood biomass is inherently more challenging than from hardwoods due to differences in composition, structure, and bonding. Softwood hemicellulose is primarily composed of galactoglucomannan (~15–20%) and arabinoglucuronoxylan (~5–10%), which are less acetylated and more strongly associated with lignin through lignin–carbohydrate complexes (LCCs) [83,86]. This reduced acetylation limits the effectiveness of simple autohydrolysis, which is commonly used for hardwoods. Instead, softwood hemicellulose extraction typically requires more severe conditions, such as steam explosion (180–220 °C), dilute acid hydrolysis, or mild alkaline extraction using NaOH [87,88]. These methods can solubilize approximately 40–70% of hemicellulose, depending on process severity and residence time.

During treatment, hemicellulose is released mainly as oligosaccharides and sugars such as mannose, glucose, and xylose, which can be further converted into biofuels or value-added chemicals like polyols and organic acids. However, excessive severity may lead to sugar degradation into inhibitors such as furfural and hydroxymethylfurfural (HMF), which negatively affect downstream fermentation [67,86,88]. Therefore, process optimization is critical to balance extraction efficiency and product quality. Overall, while softwood hemicellulose is more recalcitrant, it remains an important resource in integrated biorefinery systems.

#### 3.2.2. Softwood Cellulose

Cellulose extraction from softwood biomass focuses on isolating the highly crystalline cellulose fraction by removing lignin and hemicellulose that encase the cellulose microfibrils. Softwoods typically contain ~40–45% cellulose, which forms long, strong fibers due to their tracheid-based structure. However, the higher lignin content (~26–34%) and the predominance of condensed guaiacyl lignin make delignification more challenging than in hardwoods [18,23]. The Kraft pulping process remains the most widely used industrial method, operating at ~150–170 °C in alkaline conditions to dissolve lignin and part of the hemicellulose, producing a cellulose-rich pulp with yields of approximately 40–50% of the original biomass [65,67].

Alternative methods such as organosolv pretreatment, sulfite pulping, or alkaline peroxide treatment can improve cellulose purity and reduce residual lignin content. Pretreatment also enhances porosity and surface area, which are critical for downstream enzymatic hydrolysis, where cellulases convert cellulose into glucose. Due to residual lignin interference and enzyme inhibition, glucose yields in softwood systems are often slightly lower than in hardwoods, typically ~70–85% under optimized conditions [68,72,85]. Nevertheless, softwood-derived cellulose is highly valued for industrial applications, particularly in paper production, textile fibers, and nanocellulose materials, where long fiber length and high tensile strength are advantageous. Efficient cellulose extraction, therefore, requires optimized pretreatment to overcome lignin recalcitrance while preserving polymer integrity.

#### 3.2.3. Softwood Lignin

Lignin extraction from softwood biomass is particularly challenging due to the highly condensed and crosslinked nature of softwood lignin, which is composed almost exclusively of guaiacyl (G) units derived from coniferyl alcohol. This structure leads to a dense three-dimensional network with strong carbon–carbon linkages, making it more resistant to chemical cleavage compared to hardwood lignin [22,32]. Softwoods typically contain ~26–34% lignin, which must be effectively removed or modified to access carbohydrate fractions. In the kraft pulping process, lignin is fragmented and solubilized into black liquor under strongly alkaline conditions, from which it can be recovered through acid precipitation. However, kraft lignin is often chemically altered, containing sulfur and exhibiting partial condensation, which may limit its reactivity for certain applications [67,89].

To obtain higher-quality lignin, organosolv processes are increasingly employed, using organic solvents (e.g., ethanol or acetone) at ~160–200 °C to extract lignin in a relatively unmodified, sulfur-free form. Recovery yields are typically ~50–65% of native lignin, depending on process severity [70,89]. Extracted lignin is a valuable renewable aromatic resource and can be converted into phenolic resins, adhesives, carbon fibers, polyurethane precursors, and fine chemicals. In thermochemical pathways such as pyrolysis and gasification, softwood lignin contributes significantly to biochar formation and aromatic-rich bio-oil, reflecting its high carbon content and thermal stability. Modern biorefinery approaches increasingly emphasize “lignin-first” strategies to preserve lignin structure and maximize its valorization potential alongside carbohydrate utilization.

Instantly, the unique chemical and structural characteristics of bark, sapwood, and heartwood dictate their potential for various biomass conversion processes. Bark is better suited for thermochemical applications since it is high in lignin, extractives, and minerals. Because sapwood has a reduced extractive concentration and more accessible carbohydrates, it promotes fermentable sugar generation and biochemical conversion. Heartwood, on the other hand, has more condensed lignin and extractives, which increase durability while decreasing permeability and enzymatic digestion. Because of their condensed guaiacyl lignin structure, softwoods often exhibit higher lignin content and more recalcitrance than hardwoods. For effective biomass valorization and integrated biorefinery development, fraction-specific processing techniques are therefore essential. 

## 4. Applications of Lignocellulosic Polymers for Biofuel Production and Advanced Valorization

Lignocellulosic polymers derived from woody biomass represent important renewable feedstocks for sustainable biofuel production and biorefinery applications (Table 5). Cellulose and hemicellulose can be hydrolyzed into fermentable sugars and subsequently converted into bioethanol, biobutanol, and other bio-based chemicals through microbial fermentation. In addition, thermochemical conversion processes such as pyrolysis, gasification, and hydrothermal liquefaction enable the production of bio-oil, syngas, biochar, and hydrogen-rich fuels, particularly from lignin-rich fractions such as bark and heartwood. Lignin, owing to its aromatic structure and high carbon content, also serves as a promising precursor for carbon materials, phenolic resins, adhesives, and high-value aromatic chemicals. Beyond biofuel production, plant-derived polymers have attracted increasing interest for advanced applications, including biodegradable materials, nanocellulose composites, sustainable catalysts, and renewable platform chemicals. These emerging valorization pathways further support the development of integrated lignocellulosic biorefineries and circular bioeconomy systems.

### 4.1. Hemicellulose

Wood hemicellulose is a heterogeneous, amorphous polysaccharide primarily composed of xylans in hardwoods and glucomannans in softwoods (Table 5). Functionally, it surrounds cellulose microfibrils and covalently links with lignin, creating a complex matrix that governs biomass recalcitrance. In biorefineries, hemicellulose is preferentially solubilized during hydrothermal, dilute acid, or steam explosion pretreatments, yielding a C_5_-rich sugar stream—predominantly xylose—that comprises 50–70% of the hemicellulose fraction depending on the wood species [45,68]. While hemicellulose constitutes 20–35% [1,18] of total dry biomass and achieves 70–90% conversion efficiency to fermentable sugars under optimized pretreatment, downstream ethanol fermentation remains a bottleneck [47,85]. Yields are often constrained to 40–70% of the theoretical maximum due to the formation of degradation inhibitors (e.g., furfural, acetic acid) and the limitations of native microbial C_5_-metabolism [90]. To address this, industrial advancements utilize engineered yeasts and bacteria capable of C_5_/C_6_ co-fermentation, which enhance overall ethanol yields by 10–25% over native strains [10,16].

### 4.2. Cellulose

Wood cellulose is a linear, highly crystalline homopolymer of β-1,4-glucan units, representing the dominant fermentable fraction in most lignocellulosic feedstocks (Table 3). It typically constitutes 35–50% of dry wood biomass, depending on the species [46,87]. Industrial conversion initiates with pretreatment to disrupt the crystalline structure and alleviate lignin shielding, followed by enzymatic saccharification using tailored cellulase cocktails. Under optimized commercial conditions, enzymatic hydrolysis achieves glucose yields of 80–95% [85], while subsequent glucose-to-ethanol fermentation reaches 85–95% of the theoretical maximum using robust industrial *Saccharomyces cerevisiae* strains [91,92]. Consequently, overall wood-to-ethanol conversion yields in second-generation (2G) biorefineries range between 250 and 400 L of ethanol per metric ton of dry biomass, dictated by feedstock composition and process integration. Important thermochemical pretreatments, such as steam explosion and dilute acid processing, remain industry standards due to their favorable balance between operational expenditure and sugar recovery. Commercial-scale facilities leverage these integrated configurations to push total carbohydrate (cellulose and hemicellulose) utilization efficiencies to 70–85% of total available sugars, significantly outperforming first-generation starch-based systems in fuel yield per hectare.

### 4.3. Lignin

Wood lignin is an amorphous, highly cross-linked aromatic heteropolymer composed of phenylpropanoid units (Table 3)—specifically guaiacyl (G), syringyl (S), and p-hydroxyphenyl (H) monomers—typically constituting 15–30% of dry wood biomass [18,46]. While imparting structural rigidity and hydrophobicity to the plant cell wall, its complex network is highly recalcitrant to biochemical degradation. Unlike structural carbohydrates, lignin is non-fermentable to ethanol; instead, its higher heating value (HHV) of approximately 23–26 MJ/kg makes it a potent solid fuel and a valuable precursor for targeted thermochemical and catalytic upgrading [93,94]. Industrial valorization pathways include fast pyrolysis, which yields 60–75 wt% bio-oil from lignin-dense fractions under optimized conditions, and gasification, achieving a syngas cold gas efficiency of 60–80% [95]. Alternatively, hydrothermal liquefaction (HTL) generates 40–65% biocrude yields depending on catalyst selection and process parameters [46,96], though subsequent hydrodeoxygenation (HDO) losses reduce final liquid fuel yields to a net efficiency of 30–50%. Beyond bulk fuel production, advanced catalytic depolymerization via optimized hydrogenolysis pathways cleaves robust inter-unit linkages to recover aromatic monomers at 20–40% yields, offering a viable route to renewable phenolics and specialty chemicals [42,55,97].

## 5. Challenges and Future Directions

Despite significant advances in lignocellulosic biomass valorization, several challenges remain in efficiently extracting and utilizing polymers from wood fractions. One of the primary limitations is biomass recalcitrance, arising from the complex and highly ordered structure of cellulose, hemicellulose–lignin interactions, and the presence of lignin–carbohydrate complexes (LCCs), which hinder chemical and enzymatic accessibility [17,26,66]. This challenge is particularly pronounced in softwoods, where condensed guaiacyl-type lignin increases resistance to delignification and reduces overall conversion efficiency. Additionally, process severity trade-offs remain a critical issue: harsh pretreatment conditions improve polymer accessibility but often lead to sugar degradation and the formation of inhibitory byproducts such as furfural and phenolic compounds, negatively affecting downstream fermentation [85,98].

Another crucial challenge is the heterogeneity of biomass, including variations between hardwood and softwood species, as well as among bark, sapwood, and heartwood fractions. These differences complicate process standardization and limit the scalability of biorefinery technologies. Furthermore, current industrial processes often prioritize cellulose utilization, while lignin remains underutilized, frequently burned for energy rather than upgraded into high-value products [65,99]. Future research should focus on developing integrated and selective fractionation technologies, such as organosolv and “lignin-first” approaches, which enable simultaneous recovery of high-quality hemicellulose, cellulose, and lignin. Advances in green solvents, ionic liquids, and deep eutectic solvents also offer promising pathways for more sustainable and efficient biomass processing. In addition, improving enzyme engineering and microbial tolerance will enhance conversion yields and reduce process costs [30,87]. Ultimately, a holistic biorefinery strategy that integrates feedstock-specific optimization, process intensification, and full biomass valorization will be essential to advance the economic and environmental viability of lignocellulosic biofuels and bio-based materials.

## 6. Conclusions

This review highlights the critical importance of understanding the compositional and structural differences between hardwood and softwood lignocellulosic biomass, as well as among their anatomical fractions—bark, sapwood, and heartwood. These variations significantly influence polymer accessibility, extraction efficiency, and suitability for different conversion pathways. Sapwood emerges as the most favorable fraction for biochemical conversion due to its high cellulose and hemicellulose accessibility and low extractive content. In contrast, bark and heartwood, characterized by higher lignin and extractive concentrations, are better suited for thermochemical processes and the recovery of high-value bioactive compounds. Softwoods generally exhibit greater recalcitrance than hardwoods due to their condensed guaiacyl lignin structure, requiring more intensive pretreatment strategies. Advances in fractionation technologies, such as organosolv and lignin-first approaches, offer promising opportunities to improve the selective recovery of biomass components while preserving their functionality. However, challenges related to biomass heterogeneity, process optimization, and inhibitor formation remain significant barriers to large-scale implementation. Future research should focus on integrated biorefinery systems that combine feedstock-specific optimization with sustainable processing technologies. Such approaches will be essential to maximize resource efficiency, reduce reliance on fossil fuels, and support the transition toward a circular bioeconomy.

## Figures and Tables

**Table 1:** Main chemical components and structural characteristics of lignocellulosic biomass.

Feature/Component	Hemicellulose	Cellulose	Lignin
General Classification	Heterogeneous, amorphous, branched polysaccharides.	Linear homopolysaccharide, (C_6_H_10_O_5_)_n_.	Complex, 3D phenylpropanoid polymer.
Structural Characteristics	Species-dependent.	Highly ordered microfibrils	Rigidity, hydrophobicity, resistance.
Monomeric/Repeating units	Xylose, mannose, glucose, arabinose, galactose, and uronic acids.	β-D-glucopyranose units (fundamental repeating unit: cellobiose).	Three monolignols: p-coumaryl alcohol (H-unit), coniferyl alcohol (G-unit), and sinapyl alcohol (S-unit).
Linkages/Bonding	Matrix-bound branching (varies by species).	β(1→4) glycosidic bonds, extensive hydrogen bonding.	Dominant: β-O-4 Others: β-β, β-5, 5-5
Function in cell wall	Acts as a matrix polymer, binds cellulose, and provides structural flexibility and moisture regulation.	Forms microfibrils via hydrogen bonding to provide mechanical strength and rigidity.	Interlocks the polysaccharide network, provides structural support, waterproofing, and defense.

**Table 2:** Selected Hardwood Species: Climatic Distribution, Geographic Occurrence, and Properties.

Hardwood Species	Climate Zone	Main Countries/Regions	Properties (Wood & Biomass Relevance)	References
Poplar (*Populus* spp.)	Temperate (non-tropical)	USA, Canada, China, Europe	Low–moderate lignin, high cellulose, fast-growing, high enzymatic digestibility, excellent biofuel feedstock	[1,36,37]
Willow (*Salix* spp.)	Temperate	Europe, UK, China, Canada	Short-rotation crop, balanced cellulose/hemicellulose, low ash, suitable for bioenergy plantations	[38,39]
Birch (*Betula* spp.)	Temperate	Northern Europe, Russia, Canada	High xylan hemicellulose, moderate lignin, good for sugar production after pretreatment	[40,41]
Oak (*Quercus* spp.)	Temperate	USA, Europe, Asia	High lignin and extractives, dense wood, durable, better for thermochemical biofuel routes	[42,43]
Maple (*Acer* spp.)	Temperate	North America, Europe, East Asia	Balanced lignocellulose composition, moderate density, good biorefinery potential	[44,45]
Beech (*Fagus* spp.)	Temperate	Europe, USA, Asia	Uniform structure, moderate lignin, consistent processing behavior	[24,46]
Aspen (*Populus* spp.)	Temperate (cold regions)	Scandinavia, Canada, Russia	Low lignin, high cellulose accessibility, model species for biofuel research	[47,48]
Eucalyptus (*Eucalyptus* spp.)	Tropical & subtropical	Brazil, India, Australia, Africa	Fast growth, high biomass yield, variable lignin, widely used in bioenergy plantations	[49,50]
Acacia (*Acacia* spp.)	Tropical	Africa, India, Australia, Southeast Asia	Nitrogen-fixing, variable chemistry, moderate lignin, good for degraded land biomass, fast-growing plantation species, good fiber yield	[51,52]
Teak (*Tectona grandis*)	Tropical	India, Myanmar, Thailand, Indonesia, Africa	High extractives and lignin, highly durable wood, low biodegradability	[53,54]
Rubberwood (*Hevea brasiliensis*)	Tropical	Thailand, Malaysia, Indonesia	Plantation residue wood, moderate lignin, widely used for secondary biomass	[55,56]

**Table 3:** Hardwood Bark—Polymer Composition, Properties, and Representative Species.

Category	Component/Feature	Characteristics of Hardwood Bark	Representative Hardwood Species (Examples)	Biofuel/Biorefinery Relevance
Structural polymers	Cellulose	Low content; poorly organized microfibrils; low accessibility	Poplar (*Populus* spp.), Oak (*Quercus* spp.), Birch (*Betula* spp.)	Limited fermentable sugar yield unless intensive pretreatment is applied
Structural polymers	Hemicellulose	Highly heterogeneous; rich in xylans, arabinose, galactose, and uronic acids	Birch (*Betula* spp.), Maple (*Acer* spp.), Poplar (*Populus* spp.)	Source of pentose sugars (xylose) for bioethanol and biochemicals
Structural polymers	Lignin	High content; condensed and irregular; often linked with suberin and phenolics	Oak (*Quercus* spp.), Eucalyptus (*Eucalyptus* spp.), Teak (*Tectona grandis*)	High energy density; suitable for pyrolysis, gasification, biochar
Non-structural compounds	Extractives	Very high: tannins, flavonoids, phenolics, resins, waxes, suberin derivatives	Oak (tannin-rich), Birch (betulin-rich), Eucalyptus (phenolic-rich)	Source of high-value chemicals; may inhibit fermentation
Inorganic fraction	Ash/minerals	Higher than wood; includes Ca, K, Si	Species-dependent; higher in fast-growing species like Poplar, Eucalyptus	Influences slagging, ash formation, and combustion efficiency
Physical properties	Structure	Highly heterogeneous, dense, irregular tissue organization	All hardwoods, pronounced in Oak and Eucalyptus	Reduces enzymatic accessibility; requires pretreatment
Biological resistance	Decay resistance	High due to lignin + extractives + suberin	Oak (very high), Teak (extremely durable), Birch (moderate)	Enhances durability but reduces biodegradability
Biofuel suitability	Thermochemical conversion	Highly suitable due to lignin richness	Eucalyptus, Oak, Teak	Produces bio-oil, syngas, biochar
Biofuel suitability	Biochemical conversion	Limited unless strong pretreatment is applied	Poplar, Birch, Maple (more favorable than Oak/Teak)	Fermentable sugars after hydrolysis

**Table 4:** Selected Softwood Species: Climatic Distribution, Geographic Occurrence, and Properties.

Softwood Species	Climate Zone	Main Countries/Regions	Properties (Wood & Biomass Relevance)	References
Pine (*Pinus* spp.)	Temperate & boreal	USA, Canada, Europe, China	High lignin (~26–32%), rich in resin acids, moderate cellulose (~40–45%), widely used for pulp and bioenergy; relatively high recalcitrance	[12,68]
Spruce (*Picea* spp.)	Boreal (cold temperate)	Scandinavia, Russia, Canada	Uniform tracheid structure, low extractives (~1–5%), high cellulose accessibility after pretreatment, major pulpwood species	[37,68]
Fir (*Abies* spp.)	Temperate & boreal	Europe, North America, Asia	Moderate lignin (~27–30%), low resin content, good pulping behavior, relatively uniform fiber structure	[69,70]
Larch (*Larix* spp.)	Cold temperate	Russia, Northern Europe, Canada	Higher extractives (~5–10%), dense wood, relatively high lignin, and more resistant to biochemical conversion	[71,72]
Douglas-fir (*Pseudotsuga menziesii*)	Temperate	North America, Europe (plantations)	High-strength wood, lignin-rich (~28–31%), good structural biomass, moderate-to-low enzymatic digestibility	[73,74]
Hemlock (*Tsuga* spp.)	Temperate	North America, East Asia	Lower density softwood, moderate lignin (~26–29%), used in pulp industry, relatively uniform tracheid structure	[75]
Cedar (*Cedrus* spp.)	Temperate & Mediterranean	Middle East, Mediterranean, Himalayas	High aromatic extractives, natural durability, lower biodegradability, and suitable for thermochemical conversion	[76,77]
Cypress (*Cupressus* spp.)	Temperate & subtropical	Mediterranean, USA, Asia	High durability, rich in oils and extractives (~5–12%), strong resistance to microbial degradation	[78,79]
Yew (*Taxus* spp.)	Temperate	Europe, Asia, North America	Very high extractives (alkaloids), slow growth, dense and highly durable wood	[80,81]

**Table 5:** Overview of pretreatment methods, conversion efficiencies, and industrial platforms for wood biomass polymer fractions (hemicellulose, cellulose, and lignin) in lignocellulosic biorefineries.

Metric/Parameter	Hemicellulose	Cellulose	Lignin
Composition in Dry Wood	20–35%	35–50%	15–30%
Chemical Structure	Heterogeneous, amorphous branch polysaccharide (xylans/glucomannans)	Linear, highly crystalline homopolymer (β-1,4-glucose units)	Amorphous, highly cross-linked three-dimensional aromatic heteropolymer (G, S, H units)
Primary Conversion Pathway	Biochemical (Fractionation & Co-fermentation)	Biochemical (Saccharification & Fermentation)	Thermochemical & Catalytic (Pyrolysis, Gasification, HTL, Hydrogenolysis)
Primary Biofuel Product	C_5_-rich Bioethanol/Furfural	Second-generation (2G) Bioethanol	Bio-oil, Syngas, Biocrude, Aromatic Monomers
Pretreatment/Extraction	Preferential solubilization (Hydrothermal, dilute acid)	Thermochemical disruption (Steam explosion, dilute acid)	Commercial extraction protocols (LignoBoost, LignoForce)
Hydrolysis/Conversion Efficiency	70–90% sugar conversion	80–95% glucose conversion	Fast Pyrolysis: 60–75 wt% bio-oil Gasification: 60–80% syngas energy efficiency HTL: 40–65% biocrude
Downstream Fermentation Yield	40–70% of theoretical yield (constrained by inhibitors and C_5_ metabolism)	85–95% of theoretical yield (using *S. cerevisiae*)	Non-fermentable (Upgraded via hydrodeoxygenation to liquid fuels at 30–50% efficiency)
Industrial Advancements	Engineered strains for C_5_/C_6_ co-fermentation (yield boost of 10–25%)	Tailored cellulase cocktails; robust industrial yeast strains	Targeted catalytic cleavage of robust inter-unit linkages (20–40% aromatic monomers)
Commercial Benchmark Platforms	Clariant’s sunliquid^®^	Raízen	LignoBoost, LignoForce

## Data Availability

Data are contained within the article.

## Abbreviations

The following abbreviations are used in this manuscript:

G	Guaiacyl (monolignol-G)
HHV	Higher heating value
HMF	Hydroxymethylfurfural
HTL	Hydrothermal liquefaction
HWE	Hot water extraction
LCCs	Lignin–carbohydrate complexes
S	Syringyl (monolignol-S)
